# Recruiting and retaining healthcare workers in Scotland to a longitudinal COVID-19 study: a descriptive analysis

**DOI:** 10.1186/s12874-024-02380-6

**Published:** 2024-11-01

**Authors:** Josie MM Evans, Nicole Sergenson, Melanie Dembinsky, Lynne Haahr, Jen Bishop, Anna Howells, Katie Munro, Lesley Price

**Affiliations:** 1 Public Health Scotland, Glasgow, UK; 2 Glasgow Caledonian University, Glasgow, UK; 3 UK Health Security Agency, London, UK

**Keywords:** Recruitment, Retention, Health care workers, COVID-19

## Abstract

**Background:**

Rapid timescales for the design and delivery of research were common during the COVID-19 pandemic. The recruitment and retention of healthcare workers (HCWs) as participants in research studies are notoriously challenging, but this was exacerbated during the pandemic by the unprecedented demand placed on the workforce. The SARS-CoV-2 Immunity and Reinfection Evaluation (SIREN study) is a prospective multicentre cohort study following HCWs in the UK. This paper discusses the strategies and challenges associated with recruitment and retention of HCW participants in Scotland.

**Methods:**

There were 44,546 HCWs recruited to the SIREN study, of whom 6,285 were recruited by research teams at ten different research sites in Scotland between October 2020 and March 2021. Information on target and actual sample size, availability of resource, recruitment rate, and recruitment and engagement strategies by site was collated from SIREN study documentation and discussions with local key SIREN site staff. Individual-level data from 6,153 HCW participants with ongoing consent for all data usage were also collated, including socio-demographic data and information on withdrawal (in first year) and opt-in to a study extension after one year. Factors associated with these outcomes were explored in logistic regression analyses.

**Results:**

Different recruitment strategies were used in each site according to local agreements, protocol and staff capacity, with the recruitment period ranging from 13 to 160 days. The locally-agreed recruitment target was met in four sites. The proportion of participants who withdrew in the first year ranged from 3.1 to 24.8% by site, while subsequent opt-in to a 12-month study extension ranged from 28.6 to 74.8%. The sites with the highest proportions of withdrawals were the same four sites with lowest proportions of opt-in. On an individual level, there was a lower level of retention among younger participants, and those from lower socio-economic backgrounds and minority ethnic groups.

**Conclusions:**

Site-specific factors including research-readiness likely had a significant influence on recruitment and retention, more so than the specific recruitment or retention strategies employed. Independent of site factors, individual-level variables influenced recruitment and retention, suggesting targeted strategies may be needed to promote research engagement among particular socio-demographic groups.

## Background

The emergence of SARS-CoV-2, the COVID-19 pandemic and its impact at an individual, societal and policy level generated a wealth of new research questions [1]. Efforts to understand SARS-CoV-2 transmission and infection and re-infection was vital, as was a co-ordination of efforts to understand the effects of interventions [2]. In the UK alone, more than 1 million people took part in COVID-19 research in the first year of the pandemic [3]. One population group in particular demand as participants in COVID-19 research studies was healthcare workers (HCWs) [4]. HCWs had early and increased ongoing exposure to the virus, with early access to vaccines and easy access to testing compared to the general population.

The recruitment and retention of participants in health research studies is notoriously challenging [5], and there are particular challenges associated with an HCW cohort [6]. These can include staff time pressures and study burden which were exacerbated by the pandemic due to a lack of planning time, restrictions to in-person appointments, reduced staff availability, rapid changes in guidance, and high levels of uncertainty for researchers and potential research participants.

The SIREN study was established at pace and scale within this context. It is a large-scale observational opt-in study among HCWs working in hospital settings in the UK National Health Service (NHS). The original over-arching aim of the study was to determine whether the presence of antibody to SARS-COV2 (anti-SARS-COV2) is associated with a reduction in the subsequent risk of infection over short-term periods, the next year and in the longer-term [7]. The study was amended to investigate COVID-19 vaccine effectiveness in January 2021. There were 135 health care sites that agreed to recruit participants for an initial one-year period (subsequently extended) to follow a data collection protocol, involving a baseline nose and throat swab for PCR analysis, a self-report survey of demographics and history of COVID-19 infection and symptoms, and serology testing. Subsequent data collection involved two-weekly swabbing and survey of symptoms, testing, hospitalisation and vaccination, and ongoing serology testing at monthly or quarterly intervals [7], in addition to any other testing required for their role. In total, 44,546 participants were recruited to the SIREN study, of whom 6,285 were recruited in Scotland between October 2020 and March 2021, this process being co-ordinated by an independent Scottish SIREN research team.

In this paper, we present data relating to the recruitment of participants in Scotland within ten Scottish Health Board sites, and the retention of these participants over the first 12 months of the SIREN study, providing some insight into the issues and challenges around recruitment and retention to research within the NHS in Scotland.

## Methods

The SIREN study was run at ten Scottish sites, each with a Principal Investigator and local research team. Nine of these were Health Boards (with one or more district or community hospitals involved) and one was a large national specialist hospital which is managed by a specialist Health Board appointed by NHS Scotland. Once a decision to participate had been made by Health Board management, and research governance procedures and approvals were in place, each site commenced recruitment. The date of opening recruitment varied by site (earliest date 20 October 2020) but recruitment at all sites stopped on 31 March 2021. Each site had a different target sample size for recruitment, determined according to population size of the Health Board catchment area, but no site-specific incentives to reach recruitment targets. Recruitment and engagement strategies at each site were left to local research teams to devise and implement, according to availability of research staff and support, and local protocols. However, monthly forums were held when study sites came together to share good practice. HCWs were recruited from a range of job roles including clinical staff (doctors, nurses, midwives, allied health professionals), executive or administrative staff, and support staff (estates, porters and security).

For the purpose of this comparison, information on target and actual sample size, availability of resources, and recruitment rate, was collated from SIREN study documentation. A checklist was devised to gather information on availability of resources and staff, and specific recruitment and engagement strategies employed, and this was used as the basis of informal discussions with key SIREN staff at each site, at which notes were taken. These discussions were carried out by co-authors MD and NS.

Participants were initially recruited to participate in the SIREN study for one year, but they could withdraw from the study at any time. This required completion of an online questionnaire where they could provide a reason for withdrawal. After 48 weeks they were invited to extend their participation for a further year if they had not already withdrawn.

Individual-level data from 6,153 participants with ongoing consent for all data usage were collated, including information on withdrawal and extension. Demographic data were available from an online questionnaire completed by participants upon enrolment. Personal identifiers provided in the enrolment questionnaire were used to obtain Community Health Index (CHI) number, which uniquely identifies a person within NHS Scotland. SIREN study sites in Scotland report laboratory test results to a national surveillance system (Electronic Communication of Surveillance in Scotland (ECOSS)) held by Public Health Scotland (PHS). CHI number was used to link participant data to laboratory test results in ECOSS, and also to identify patient postcode in order to add Scottish Index of Multiple Deprivation (SIMD) quintile (a postcode measure of material deprivation). The SIREN study dataset also includes PCR data for tests taken from January 2020 to enrolment, and tests taken outside the fortnightly SIREN study testing.

The proportions of participants who withdrew from the SIREN study during the first year, and then of those that remained, the proportions who actively opted into the extension, were compared between socio-demographic and occupational groups. Logistic regression analyses were conducted for each of these outcome measures (withdrawal and opt-in), and odds ratios (with 95% confidence intervals) determined to assess the associations between the independent variables and the outcome. All independent variables were adjusted independently for the other variables in a multivariable analysis. Site was also included as a covariate.

## Results

### Recruitment and engagement strategies

Summary recruitment, withdrawal, and retention data are presented in Table 1 for the ten sites. Different recruitment strategies were used in each site according to local agreements, protocol and staff capacity. All sites used some form of electronic recruitment: either all-staff routine electronic communications (e.g. newsletters, bulletins, Intranet) to advertise the study, or mass study-specific emails to staff email addresses. This was usually supplemented by distribution of posters/leaflets and/or face-to-face recruitment. Some sites specifically highlighted recruitment using social media sites (Facebook and Twitter), and six out of ten sites also mentioned the importance of word-of-mouth. Sites B and D appeared to have the most limited recruitment strategies: Site B relied on electronic communication only and Site D relied on electronic recruitment combined with word-of-mouth only. However, Site B exceeded its recruitment, while Site D did not meet the target.

**Table 1:** Characteristics of participating sites

Site (no. of hospitals)	Experience of PI/research team & support infrastructure	Target sample size	Week recruitment started (total days)^1^	Final sample size (% of target)^1^	% withdrew in first year^1^	% extension^1^	Recruitment strategies	Data collection strategies	What worked well for engagement
A (1)	Lower levels of experience. Less well-staffed: Dedicated PI but research nurse support less consistent	209	16/3/21 (13)	17 (8.1%)	17.6%	28.6%	Mass email Leaflets/posters No face-to-face recruitment	Serology: Occupational health and some peer sampling PCR: Asymptomatic testing team	Wider study results and updates (including webinar links) also circulated electronically Appointment flexibility
B (1)	Some research experience, but small-scale Less well-staffed but 2 consistent staff members	66	11/3/21 (20)	67 (101.5%)	11.9%	62.7%	All-staff electronic comms Mass e mail No face-to-face recruitment	Serology: dedicated SIREN clinic and peer sampling PCR: dedicated SIREN clinic and self	Regular e mail communication from central SIREN inbox, including wider study results
C (1)	High level of experience Well-staffed Specialist hospital site	113	26/11/20 (125)	131 (115.9%)	3.1%	74.8%	All-staff electronic comms Face-to-face with ward outreach Leaflets/Posters	Serology: research centre/staff work places and very little peer sampling PCR: research centre /staff work places	Wider study results communicated via posters Regular electronic comms including messaging from dedicated study phone for results, appointment reminders and information on changes to national policy Appointment flexibility Proactive reminders to participants who had not provided a sample for a while Samples taken in research centre or work area, waiting times minimised
D (1)	High level of experience Well-staffed: relatively small but consistent team	429	31/12/20 (90)	186 (43.4%)	13.4%	61.4%	All-staff electronic comms Mass email Word of mouth No face-to-face recruitment	Self, peer sampling and research clinic	Regular feedback and updates on staff intranet (newsgrab on home page or email direct to participants) Regular acknowledgement of contribution Appointment flexibility
E (5)	High level of experience Well-staffed	602	10/12/20 (111)	404 (67.1%)	11.7%	57.5%	All-staff electronic comms Face-to-face with ward outreach Leaflets/Posters Word of mouth	Serology: research clinic and peer sampling PCR: self, peer sampling and clinic.	Wider study results circulated via staff notice board/intranet Appointment flexibility Appointment reminders
F (1)	Some research experience, but small-scale Well-staffed	619	21/1/21 (69)	449 (80.6%)	13.8%	62.0%	All-staff electronic comms Leaflets/Posters Social media Word of mouth	Serology: peer sampling and clinic PCR: self	Limited mass e mails (mainly individual) Posters Appointment reminders Appointment flexibility (coming to wards) Notification if missed a visit
G (2)	Some research experience Well-staffed: large but changing team	976	10/12/20 (111)	697 (71.4%)	11.5%	63.4%	All-staff electronic comms Leaflets/Posters Social media Word of mouth Limited face-to-face recruitment	Serology: SIREN clinic PCR: self and SIREN clinic	Individual and mass e mails Wider study results on posters and in monthly newsletter SIREN clinics welcoming and friendly Monthly newsletter containing local hospital news, SIREN papers, Long COVID papers and prize draw every month with gifts/incentives Appointment reminders Appointment flexibility
H (3)	High level of experience Well-staffed	875	14/1/21 (76)	1,096 (125.3%)	18.3%	48.3%	All-staff electronic comms Face-to-face with ward outreach Leaflets/Posters Word of mouth	Serology: SIREN clinic and peer sampling PCR: SIREN clinic and self	Wider study results communicated via posters and e mail Appointment reminders Notification if missed a visit Drop-in dedicated SIREN clinic available
J (3)	High level of experience Well-staffed	2,527	26/11/20 (125)	1,207 (47.8%)	17.8%	54.4%	All-staff electronic comms Face-to-face recruitment with outreach particularly for support staff	Peer and staff sampling services	Wider study results communicated via posters and quarterly newsletter. Closing of local clinic affected retention
K (3)	High level of experience Well-staffed	1,646	22/10/20 (160)	1,902 (115.6%)	24.8%	37.0%	All-staff electronic comms and targeted e mails Face-to-face with ward outreach Word of mouth	Serology: Mainly peer sampling, very few clinics PCR: self	Regular electronic comms including e mails (no mass e mails), messaging from dedicated study phone Appointment reminders Notification if missed a visit Drop-in available

^1^Shading where values are particularly low or high

The recruitment period ranged from only 13 days to 160 days. There were three sites (Sites A, D, J) where recruitment was below 50% of the target. Site A had a very short recruitment period and no face-to-face recruitment, Site D also had no face-to-face recruitment, while Site J had a very large target sample size. Sites B, C, H and K exceeded the target.

### Withdrawal and retention by site

The main engagement strategies used by sites were regular communication with participants, feeding back regularly to them on wider study results, flexibility of appointments, and reminders and notifications. The proportion of participants who withdrew during the first 12 months ranged from 3.1 to 24.8% by site. The four sites with the highest withdrawals included the two largest sites (Sites J, K) and the smallest site (Site A - where the sample size was very small). The proportions of those who had not already withdrawn who actively opted into the extension by site (rather than actively opting out or not replying) ranged from 28.6 to 74.8%. The sites with the lowest proportions of opt-in were the same four sites with the highest proportions of withdrawals (Sites A, H, J, K). Site K had the highest withdrawal, and had very few dedicated research clinics, relying mainly on self and peer blood sampling, in order to cope with the high numbers of participants in that site. In contrast, Site C was notable for low withdrawal and high opt-in. This site had a focus on building relationships with participants through a dedicated research centre, generating a strong SIREN research identity and community; and there was a relatively small sample size in one location. They also had a proactive approach to reminding participants of appointments if they had not attended for a while.

In regression analyses which adjusted for the individual characteristics of participants at the sites, these site patterns all persisted, with Site E also associated with low opt-in (Tables 2 and 3).

**Table 2:** Numbers of participants withdrawing in first year stratified by socio-demographic group, and results of multivariable regression analysis among 6,153 participants with withdrawal as dependent variable

	Total (*n* = 6,153)	Total withdrawn in first year (*n* = 1,117)	Odds ratio (95% confidence interval) - adjusted for all covariates	*P* value
**Age (years)**				
18–24	337	106 (31.5%)	Ref	-
25–34	1,446	363 (25.1%)	0.79 (0.61–1.03)	0.078
35–44	1,600	248 (15.5%)	0.45 (0.35–0.60)	< 0.001
45–54	1,681	239 (14.2%)	0.40 (0.31–0.53)	< 0.001
55–64	1,030	150 (14.6%)	0.41 (0.31–0.55)	< 0.001
65+	59	11 (18.6%)	0.57 (0.28–1.15)	0.116
**Gender**				
Female	5,307	985 (18.6%)	Ref	-
Male	836	130 (15.6%)	0.78 (0.63–0.96)	0.018
Other	10	2 (25.0%)	-	-
**Job role**				
Clinical	5,350	971 (18.1%)	Ref	-
Support	112	15 (13.4%)	0.80 (0.45–1.14)	0.459
Admin	532	107 (20.1%)	1.57 (1.21–2.03)	< 0.001
Other	159	25 (15.7%)	0.97 (0.61–1.55)	0.915
**Patient contact**				
No	504	71 (14.1%)	Ref	-
Yes	5,649	1,046 (18.5%)	1.35 (1.00-1.81)	0.047
**Ethnic group**				
BAME	289	52 (18.0%)	1.02 (0.74–1.40)	0.926
White	5,858	1,063 (18.41)	Ref	-
**Scottish Index of Multiple Deprivation (SIMD)**				
1 (most deprived)	625	131 (21.0%)	Ref	-
2	1,035	199 (19.2%)	0.87 (0.68–1.12)	0.290
3	1,114	197 (17.7%)	0.84 (0.65–1.09)	0.183
4	1,375	251 (18.3%)	0.89 (0.70–1.11)	0.369
5 (least deprived)	1,932	324 (16.8%)	0.79 (0.63–1.01)	0.057
Missing	72	15 (20.8%)	-	-
**Site**				
A	17	3 (17.6%)	2.07 (0.58–7.38)	0.264
B	67	8 (11.9%)	1.08 (0.49–2.38)	0.851
C	131	4 (3.1%)	0.24 (0.09–0.68)	0.007
D	186	25 (13.4%)	1.19 (0.72–1.95)	0.496
E	402	47 (11.7%)	1.04 (0.71–1.54)	0.827
F	448	62 (13.8%)	1.24 (0.86–1.78)	0.253
G	697	80 (11.5%)	Ref	-
H	1,096	201 (18.3%)	1.73 (1.30–2.30)	< 0.001
J	1,206	215 (17.8%)	1.64 (1.24–2.17)	< 0.001
K	1,903	472 (24.8%)	2.33 (1.80–3.02)	< 0.001

**Table 3:** Numbers of participants actively opting into extension stratified by socio-demographic group, and results of multivariable regression analysis among 5,036 participants with opting-in as dependent variable

	Total *n* = 5,036 (still in study after 1 year)	Total who actively opted into extension (*n* = 2,569)	Odds ratio (95% confidence interval) - adjusted for all covariates	*P* value
**Age (years)**				
18–24	231	36 (15.6%)	Ref	-
25–34	1,083	358 (33.1%)	2.52 (1.71–3.70)	< 0.001
35–44	1,352	661 (48.9%)	4.81 (3.29–7.02)	< 0.001
45–54	1,442	918 (63.7%)	9.20 (6.29–13.44)	< 0.001
55–64	880	564 (64.1%)	8.93 (6.04–13.19)	< 0.001
65+	48	32 (66.7%)	9.65 (4.66–20.01)	< 0.001
**Gender**				
Female	4,322	2,233 (51.7%)	Ref	-
Male	706	335 (47.5%)	0.97 (0.82–1.16)	0.763
Other	8	4 (50.0%)	-	-
**Job role**				
Support	97	41 (42.3%)	0.59 (0.38–0.93)	0.022
Admin	425	275 (64.7%)	1.41 (1.10–1.80)	0.007
Clinical	4,379	2,180 (49.8%)	Ref	-
Other	134	73 (54.5%)	1.08 (0.74–1.59)	0.674
**Patient contact**				
No	433	260 (60.0%)	Ref	-
Yes	4,603	2,309 (50.2%)	1.00 (0.79–1.27)	0.992
**Ethnic group**				
BAME	237	85 (35.9%)	0.52 (0.38–0.70)	< 0.001
White	4,795	2,483 (51.8%)	Ref	-
**Scottish Index of Multiple Deprivation (SIMD)**				
1 (most deprived)	494	226 (45.7%)	Ref	-
2	836	388 (46.4%)	1.04 (0.82–1.33)	0.730
3	917	458 (50.0%)	1.10 (0.86–1.39)	0.453
4	1,124	577 (51.3%)	1.13 (0.89–1.42)	0.313
5 (least deprived)	1,608	895 (55.7%)	1.41 (1.14–1.77)	0.002
Missing	57	25 (43.9%)	-	-
**Site**				
A	14	4 (28.6%)	0.15 (0.05–0.50)	0.002
B	59	37 (62.7%)	0.78 (0.43–1.41)	0.413
C	127	95 (74.8%)	1.86 (1.19–2.93)	0.007
D	161	99 (61.5%)	0.76 (0.52–1.10)	0.149
E	355	204 (57.9%)	0.64 (0.49–0.87)	0.003
F	386	239 (61.9%)	0.79 (0.60–1.05)	0.099
G	417	391 (93.8%)	Ref	-
H	894	432 (48.3%)	0.48 (0.38–0.61)	< 0.001
J	991	539 (54.4%)	0.69 (0.56–0.86)	0.001
K	1,431	529 (37.0%)	0.35 (0.28–0.43)	< 0.001

### Withdrawal and retention in individuals

From an analysis of 1,721 responses from participants who withdrew from the SIREN study at any time, the most common reason for withdrawal was ‘workload and work commitments’, accounting for 804 (46.7%) of withdrawals. The next most common reasons were leaving employment (274; 15.9%), medical reasons (200; 11.6%) and logistical issues with attending appointments (96; 5.6%). There were only 153 (8.9%) responses that related directly to the protocol or to SIREN processes: 55 participants found the frequency of testing too high, 36 had difficulties accessing testing*/* booking appointments, and 32 disliked the testing methods.

The number of participants who withdrew in the first year (and who consented to individual data being analysed) was 1,117 (18.2%). Table 2 presents a logistic regression analysis with withdrawal in this group as the dependent variable, and adjusted odds ratios for covariates. These indicate the strength of any association between independent variables and the outcome, after adjusting for confounding effects of the other covariates (including site). Withdrawal was higher in the two youngest age groups, with nearly one-third of 18–24 year olds withdrawing in the first year, and this was confirmed in the regression analysis. There was also a relatively high proportion (20.1%) of administrative staff withdrawing compared to other staff, with an adjusted odds ratio of 1.57 (95% CI 1.21–2.03), compared to clinical staff (the reference group). Participants with direct patient contact also were more likely to withdraw, with an adjusted odds ratio of 1.35 (1.00–1.81), compared to those who had no such contact. Site effects were still evident after adjusting for individual-level effects, with Sites H, J and K independently associated with high withdrawal.

Among 5,036 participants remaining in the study after one year, 2,569 (51.0%) actively opted into the study extension. Table 3 presents a logistic regression analysis with retention as the dependent variable. Proportions of younger people opting in were relatively low (only 15.6% of 18–24 year olds, compared to 67.7% of 65 + year olds), and this association was confirmed in the regression analysis. The proportion of participants from minority ethnic groups who opted in was also low (35.9%), with an odds ratio of 0.52 (95% CI 0.38–0.70) compared to white participants (the reference group). Administrative staff (64.7%) had relatively high opt-in, with an odds ratio of 1.41 (95% CI 1.10–1.80) compared to clinical staff. There was a trend with increased opt-in with lessening deprivation. Sites A, C E, H, J and K were independently associated with low opt-in.

## Discussion

This study has provided insight into the challenges and successes of recruiting and retaining a cohort of HCWs to a large observational research study. To recruit more than 6,000 HCWs to a relatively onerous and invasive data collection protocol in just 6 months, is testament to the dedication and commitment of the entire research team and the HCW participants themselves, especially given recognised challenges in recruitment of this group [6]. It may be that a higher risk perception to COVID-19 among HCWs [8] was a motivating factor to enrol in COVID-19 research.

Each individual site managed recruitment independently but although the recruitment strategies used did not appear to differ markedly between sites, there was variable success in meeting recruitment targets. Six sites specifically mentioned the success of word-of-mouth recruitment. This was not necessarily anticipated at the outset but may be worth formalising as a recruitment approach in future studies, as has been done successfully elsewhere [9]. There was some evidence that face-to-face recruitment and ward outreach enhanced recruitment to SIREN, with two sites without face-to-face recruitment (Sites A and D) performing less well. So, while electronic communication can reach larger numbers of potential participants, supplementing with face-to-face strategies may make a considerable difference to final recruitment numbers, as has been observed in clinical trial recruitment [10].

The length of the active recruitment period was probably more of a limiting factor on recruitment success than the particular strategies used, with the site with the lowest recruitment having only two weeks for recruitment, but there were still other factors at play. Site B was an island site and had a very brief recruitment period but exceeded its (small) target sample size despite having no face-to-face recruitment, and this could possibly be explained by more rapid and effective networking across a smaller staff group. Similarly, Site C exceeded its recruitment target, and this might be partially attributable to a shared identity across a smaller staff group in one hospital location. But these two sites were different in nature from the other sites, which were all mainland Health Boards.

The other two sites that exceeded their recruitment targets were large Boards, with experience and capacity for research. Site K was able to start recruitment promptly and to put agreements and processes in place early, while Site H was able to mobilise additional research staff and dedicate resources to recruitment over a shorter time period. It is difficult to draw general conclusions, but it appears that the wider context, and also the research-readiness of the site, probably had considerable influence on recruitment success. Indeed, the fragmented approach of research in Scotland’s NHS needs to be addressed to ensure equity in access to research and representativeness across Scotland [11].

Although the engagement strategies used by sites mainly centred around regular communication and feedback to participants, and appointment flexibility and reminders, there was variation between sites as to how these were operationalised. Engagement was assessed in terms of withdrawal and actively opting in for a second year. The sites with the highest proportions of withdrawals were the same four sites with lowest proportions of opt-in for extension, suggesting that similar factors influenced these two measures. Of note is that there was low retention in three sites that were the first, second and third largest in terms of sample size. There may be a trade-off between an individualised approach that has been identified as important in retaining participants to clinical trials [12], and managing large numbers of research participants, with the contrasting experiences of Sites C and K exemplifying this. The regression models confirm that site effects cannot be explained by individual characteristics of participants at the sites. However, it is difficult to identify the reasons or mechanisms as to how differences between sites would influence withdrawal and opt-in, without more detailed and specific information on site characteristics, and this is a limitation of our analysis.

The most common reason participants themselves gave for withdrawal from SIREN was work or work commitments, and while relatively few cited site-specific factors or protocol-related reasons, such as frequency of testing (although not necessarily unrelated to work commitments), flexibility and accessibility for research participants should always be a high priority. In general, the contribution of study staff in terms of attitudes to and the valuing of research participants, communication and knowledge, should not be underestimated [13].

On an individual level, there was a high level of withdrawal and low level of opt-in to the extension among younger participants, Conversely, there was high retention of older participants. This contrasts with the low recruitment and retention to health research that has been observed for older people in the general population [14] and may again be related to higher risk perception of COVID-19 in older people, although the evidence for this is not conclusive [8]. The lower retention of people from lower socio-economic backgrounds and from ethnic minority groups has been observed elsewhere [15]. Interestingly, there was a relatively high proportion of administrative staff who withdrew from the study during the first year, but of those that stayed, there was a high proportion opting into the extension. Having no patient contact was also associated with high retention.

## Conclusions

These analyses demonstrate site level and individual level effects on recruitment and engagement to a large cohort study of HCWs in Scotland, offering learning for future studies. We have shown that there may be levelling-up required between Health Boards in Scotland to ensure comparability of research opportunity and access. We have also demonstrated a possible tension between having large sample sizes of research participants at research sites, and still being able to maintain the individualised approach that can promote continued research engagement. Finally, given that individual socio-demographic characteristics are also associated with research engagement, targeted strategies may be needed to retain engagement among under-represented socio-demographic groups to ensure that research findings are representative and relevant.

## Data Availability

Data for this paper are available upon reasonable request to the corresponding author.

## References

[1] LSHTM staff writer. On the front line: a global response to COVID-19. 2022. https://www.lshtm.ac.uk/research/research-action/features/front-line-global-response-covid-19 Accessed 08/11/2022.

[2] Haleem A, Javaid M, Vaishya R, Deshmukh SG. (n.d.). Areas of academic research with the impact of COVID-19. Am J Emerg Med. 2020;38:1524–6. 32317202 10.1016/j.ajem.2020.04.022 PMC7158762

[3] National Institute for Health Care Research. UK COVID-19 research passes one million participants. 2021. https://www.nihr.ac.uk/news/uk-covid-19-research-passes-one-million-participants/27215 Accessed 18/05/2023.

[4] Shaukat N, Ali DM, Razzak J. Physical and mental health impacts of COVID-19 on healthcare workers: a scoping review. Int J Emerg Med. 2020;13:40. 10.1186/s12245-020-00299-5. 32689925 10.1186/s12245-020-00299-5 PMC7370263

[5] Abshire M, Dinglas VD, Cajita MIA, et al. Participant retention practices in longitudinal clinical research studies with high retention rates. BMC Med Res Methodol. 2017;30:17. 10.1186/s12874-017-0310-z. 10.1186/s12874-017-0310-z PMC5319074 28219336

[6] Browne S, et al. Reflections on recruiting healthcare professionals as research participants: learning from the ONSPres Study. HRB Open Res. 2022;5:47. 36091186 10.12688/hrbopenres.13499.1 PMC9428496

[7] SIREN protocol. Impact of detectable anti-SARS-CoV-2 on the subsequent incidence of COVID-19 in 100,000 healthcare workers: do antibody positive healthcare workers have less reinfection than antibody negative healthcare workers? | medRxiv.

[8] Cipolletta S, Andreghetti GR, Mioni G. Risk perception towards COVID-19: a systematic review and qualitative synthesis. Int J Environ Res Public Health. 2022;19:4649. 35457521 10.3390/ijerph19084649 PMC9028425

[9] Botton CE, Santos LP, Moraes BG, Monteiro RB, Gomes MLB, Wilhelm EN, Pinto SS, Umpierre D. Recruitment methods and yield rates in a clinical trial of physical exercise for older adults with hypertension—HAEL study: a study within a trial. BMC Med Res Methodol. 2022;22:42. 35144532 10.1186/s12874-022-01535-7 PMC8830978

[10] Brogger-Mikkelsen M, Ali Z, Zibert JR, Andersen AD, Thomsen SF. Online patient recruitment in clinical trials: systematic review and meta-analysis. J Med Internet Res. 2020;22:e22179. 33146627 10.2196/22179 PMC7673977

[11] Macgill J. (2022). Research strategy for Scotland’s NHS. https://healthandcare.scot/default.asp?page=story&story=3056

[12] Brueton V. Retaining trial participants: an individualised approach is needed. Br Med J 2022; 376. 10.1136/bmj.o115 35039311

[13] Desai M. Recruitment and retention of participants in clinical studies: critical issues and challenges. Perspect Clin Res. 2020;11:51–3. 32670827 10.4103/picr.PICR_6_20 PMC7342339

[14] Forsat ND, Palmowski A, Palmowski Y, Boers M, Buttgereit F. Recruitment and retention of older people in clinical research: a systematic literature review. J Am Geriatr Soc. 2020;68:2955–63. 33075140 10.1111/jgs.16875

[15] Bonevski B, Randell M, Paul C, Chapman K, Twyman L, Bryant J, Brozek I, Hughes C. Reaching the hard-to-reach: a systematic review of strategies for improving health and medical research with socially disadvantaged groups. BMC Med Res Methodol 2014; 42. 10.1186/1471-2288-14-42 PMC3974746 24669751

